# Comparing Manual and Automated Spatial Tracking of Captive Spider Monkeys Using Heatmaps

**DOI:** 10.3390/ani15203056

**Published:** 2025-10-21

**Authors:** Silje Marquardsen Lund, Frej Gammelgård, Jonas Nielsen, Laura Liv Nørgaard Larsen, Ninette Christensen, Sisse Puck Hansen, Trine Kristensen, Henriette Høyer Ørneborg Rodkjær, Shanthiya Manoharan Sivagnanasundram, Bianca Østergaard Thomsen, Sussie Pagh, Thea Loumand Faddersbøll, Cino Pertoldi

**Affiliations:** 1 Department of Chemistry and Bioscience, Aalborg University, Frederik Bajers Vej 7H, 9220 Aalborg, Denmark; frga@aalborgzoo.dk (F.G.); joni@aalborgzoo.dk (J.N.); llnl23@student.aau.dk (L.L.N.L.); nchr23@student.aau.dk (N.C.); spha23@student.aau.dk (S.P.H.); tkri23@student.aau.dk (T.K.); hrodkj23@student.aau.dk (H.H.Ø.R.); ssivag23@student.aau.dk (S.M.S.); both23@student.aau.dk (B.Ø.T.); sup@bio.aau.dk (S.P.); cp@bio.aau.dk (C.P.); 2 Aalborg Zoo, Mølleparkvej 63, 9000 Aalborg, Denmark; tlf@aalborgzoo.dk

**Keywords:** *Ateles fusciceps*, pose estimation, SLEAP, ZooMonitor, animal welfare, machine learning

## Abstract

**Simple Summary:**

Zookeepers and researchers often monitor welfare by recording how animals use their enclosures, as space use and activity levels provide insights into whether animals are stimulated and engaged. Traditionally, this is performed by manually observing and recording positions, but this can be slow and prone to bias. In this study, we tested whether pose estimation, using a tool called SLEAP, could automatically track two spider monkeys and produce comparable results. We compared manual observations with automated tracking by generating heatmaps of space use and measuring time spent being active. Both methods showed strong agreement, with the monkeys spending most of their time around climbing structures. Automated tracking, therefore, offers a reliable way to save time and improve the consistency of welfare monitoring in zoological institutions.

**Abstract:**

Animal welfare assessments increasingly aim to quantify enclosure use and activity to support naturalistic behavior and improve Quality of Life (QoL). Traditionally, this is achieved through manual observations, which are time-consuming, subject to observer bias, and limited in temporal resolution due to short observation periods. Here, we compared manual tracking using ZooMonitor with automated pose estimation (SLEAP) in a mother–son pair of black-headed spider monkeys (*Ateles fusciceps*) at Aalborg Zoo. We collected manual observations on six non-consecutive days (median daily duration: 62 min, mean: 66 min, range: 52–90 min) and visualized this as spatial heatmaps. We applied pose estimation to the same video footage, tracking four body parts to generate corresponding heatmaps. Across most days, the methods showed strong agreement (overlap 83–99%, Pearson’s r = 0.93–1.00), with both highlighting core activity areas on the floor near the central climbing structures and by the door with feeding gutters. Both methods also produced comparable estimates of time spent being active, with no significant difference across days (*p* = 0.952). Our results demonstrate that computer vision technology can provide a reliable and scalable tool for monitoring enclosure use and activity, enhancing the efficiency and consistency of zoo-based welfare assessments while reducing reliance on labor-intensive manual observations.

## 1. Introduction

Black-headed spider monkeys (*Ateles fusciceps* Gray, 1866), hereafter referred to as spider monkeys, are diurnal, arboreal primates inhabiting the rainforests of Colombia, Ecuador and Panama [1,2]. They exhibit a fission–fusion social structure, forming dynamic subgroups during the day to reduce competition for food [3,4]. They are omnivorous and primarily feed on ripe fruits and supplement with leaves, insects and other foods [2,5]. Spider monkeys are listed in the Convention on International Trade in Endangered Species’ Appendix II [6]. This designation indicates that, while this species is not currently at risk of extinction, it is vulnerable to the impacts of unregulated trade [7], and the International Union for Conservation of Nature categorizes this species as endangered [1,8].

In response to conservation and welfare goals, zoological institutions, hereafter referred to as zoos, increasingly seek to promote natural behaviors and activity patterns and stimulate full enclosure use [9,10]. Assessing enclosure use reveals how animals engage with the space and resources available to them. Uneven space use or limited locomotor activity can indicate husbandry or design issues that constrain welfare, whereas diverse movement patterns and balanced enclosure use are linked to more positive welfare states [10,11]. Quality of Life (QoL) assessments similarly emphasize the importance of monitoring locomotion, exploration and the use of enclosure features as indicators of behavioral health and engagement, since both inactivity and stereotypic pacing may signal compromised welfare [9]. Together, these approaches highlight that activity and enclosure use are not only measures of physical health and stimulation but also vital indicators of whether zoo environments are enabling animals to thrive. However, these assessments often rely on behavioral observations, through video footage or live observations, but such assessments are time-consuming, subjective, and difficult to scale across numerous individuals [11,12,13].

Machine learning (ML) in the form of computer vision technology, particularly pose estimation, can offer promising solutions for automated, standardized behavior monitoring [12,14]. These methods can reduce observer bias, increase temporal resolution, and allow continuous data collection even in multi-animal settings [12,15]. Pose estimation uses trained neural networks to locate defined body parts within each video frame, generating spatial coordinates that can be used to reconstruct postures and movements [14,16]. Tools such as SLEAP, DeepLabCut and others have demonstrated high accuracy in both laboratory and field settings, enabling fine-scale quantification of behavior, activity tracking, and space use [14,16,17,18]. However, automated approaches depend on stable camera placement and sufficient visibility, which can create blind spots if not optimized. These approaches are increasingly recognized as valuable for welfare monitoring, as they provide both spatial and postural data that can inform enrichment and enclosure design [12,15,16].

In this study, we compare manual enclosure-use tracking and automated pose estimation to evaluate spatial activity in a mother–son pair of spider monkeys at Aalborg Zoo (Denmark). By assessing the overlap between methods, we explore the potential of computer vision for improving the efficiency and consistency of welfare monitoring.

## 2. Materials and Methods

Two spider monkeys housed at Aalborg Zoo were observed for this study: a female born in 1997 and her male offspring born in 2009. Observations focused exclusively on the indoor enclosure due to camera limitations. The indoor enclosure (approx. 97 m^2^) included climbing structures such as a central tree, five wooden shelves, fire hoses, and ropes. Visitor viewing was separated by netting, vegetation, and fencing.

We collected video data on six non-consecutive days between 26 October and 16 November 2024, in three sessions a day around feeding time during the zoo’s off-season. The total daily filming duration ranged from 52 to 90 min (median: 62 min). We positioned a wide-lens camera (Kitvision Escape HD5 and 4 KW (Christchurch, Dorset, BH23 4FL, UK)) to provide the most optimal coverage of the indoor enclosure.

Six observers carried out the manual observations, using the program ZooMonitor (version 4.1) [19]. Each observation session was scored by a subset of observers, and not all observers recorded data for all dates. To ensure consistency in scoring, a concordance test was conducted prior to data collection, following Altmann (1974), with a minimum agreement threshold of 85% [20]. We recorded the animals’ location using interval focal sampling [20] in 12 s intervals on a top-down schematic drawing of the enclosure, resulting in approximately 260–450 possible locations recorded per day per focal. The 12 s interval was chosen to balance temporal resolution and feasibility, based on pilot testing. A heatmap was produced from observations. We denoted coordinates for the focal sampling using a custom 2D Habitat Map in ZooMonitor and then visualized as heatmaps using R (version 4.4.3) [21].

**Figure 1:**
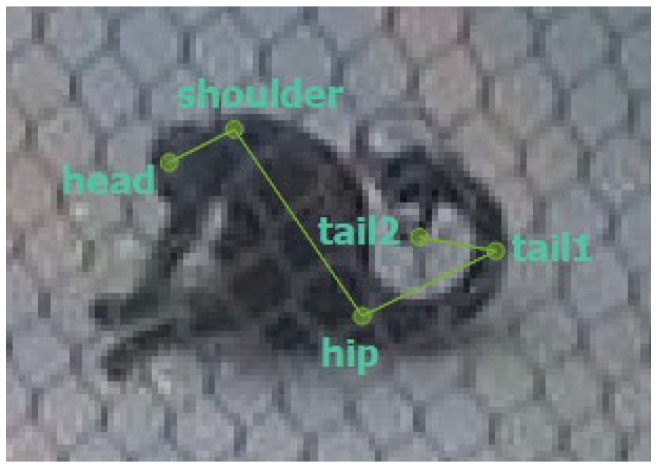
Example from a labeled frame from SLEAP showing the skeleton used for training with the 5 nodes: head, shoulder, hip, tail1 and tail2.

To compare manual scoring with automated approaches, we applied computer vision using pose estimation. Pose estimation involves training a neural network to detect and track specific body parts of the animals in each video frame, thereby generating coordinates that can be used to reconstruct their movements and enclosure use. For this purpose, SLEAP (version 1.4.1a2) [14] was used to create a model trained on 313 frames from the footage collected on 26 October 2024. We trained the model to recognize four body parts to track the individuals. This was performed with the use of a skeleton with five nodes: head, shoulder, hip, tail1, and tail2 (Figure 1). We built and trained the model using SLEAP’s prediction-assisted labeling workflow until the model consistently produced accurate predictions across multiple frames and diverse conditions. The final best model was reached after 40 epochs with a validation loss of 0.000745. We reduced the frame rate of the videos to 1 fps and converted them to grayscale for analysis, resulting in approximately 3120–5400 possible locations recorded per day per focal. We used the resulting pose data to generate heatmaps for comparison with manual scoring. We produced these heatmaps in R [21] using the following packages: tidyverse (version 2.0.0) [22], ggpointdensity (version 0.2.0.9000) [23], and jpeg (version 0.1-11) [24].

Furthermore, we calculated the daily active time for both methods. In ZooMonitor, we considered consecutive 12 s samples as “movement” if x–y changes exceeded 7 pixels. In SLEAP, we reduced each frame pose to the best-scored hip/shoulder point, with movement defined as changes > 30 pixels. For each method, the durations of movement were summed. We compared the methods using a paired *t*-test.

## 3. Results

**Figure 2:**
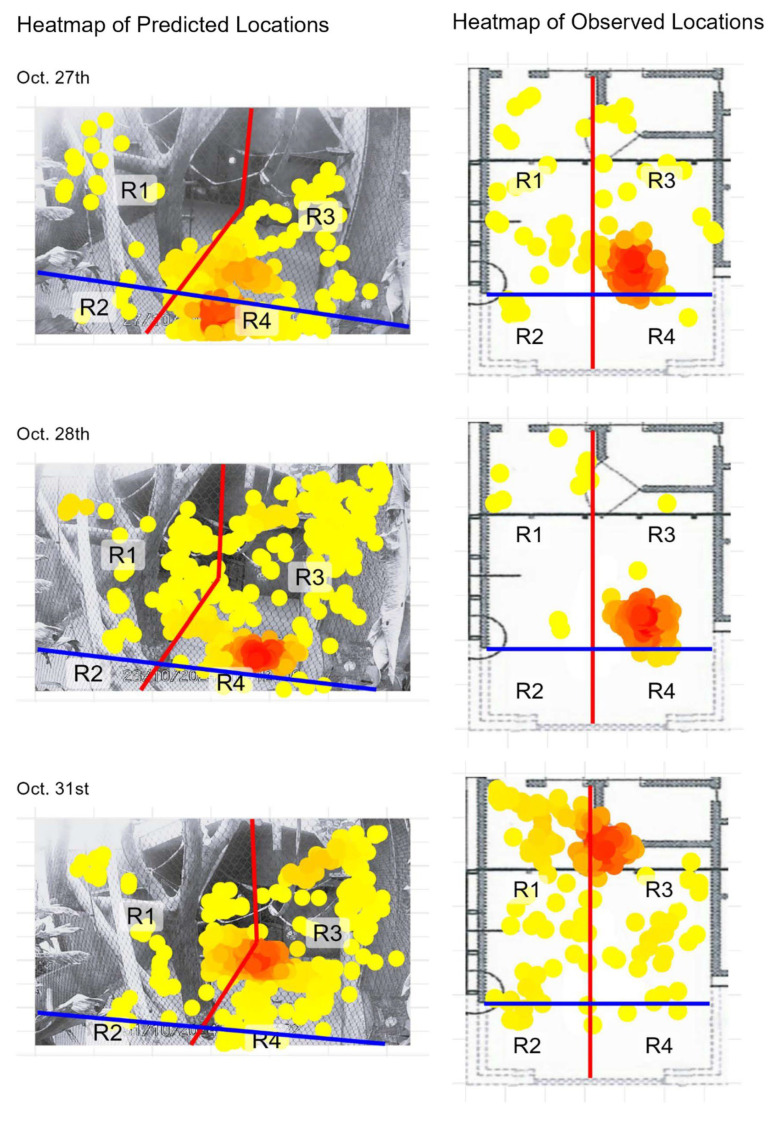

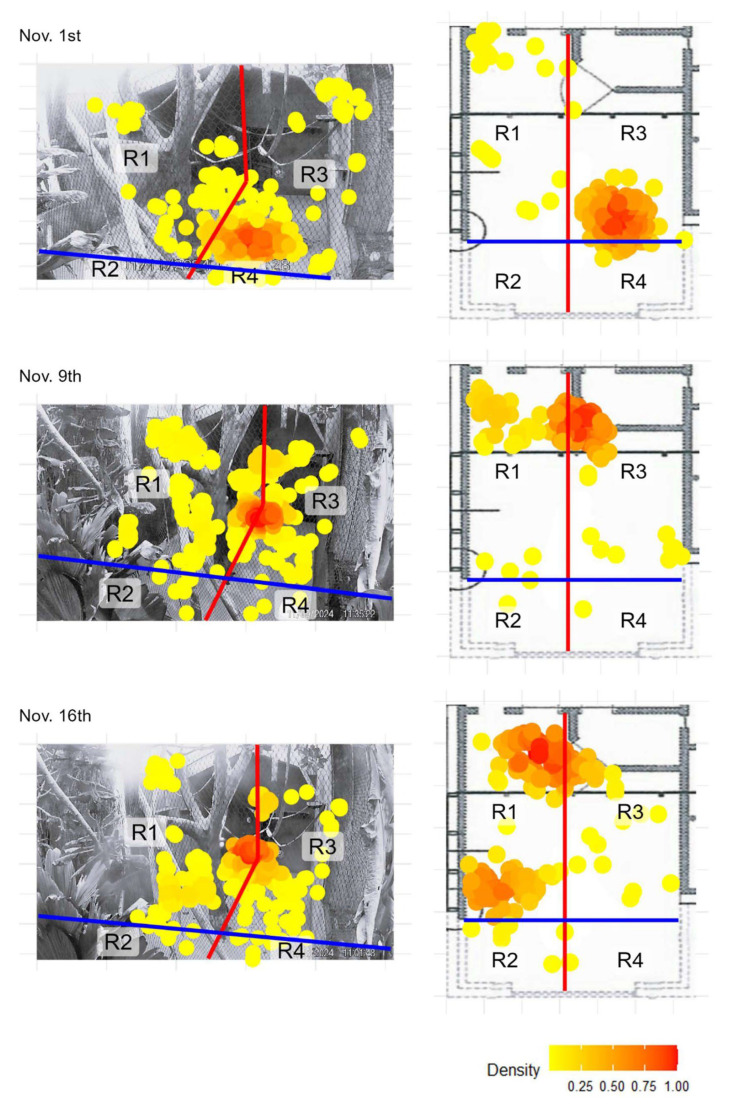
Heatmaps of model-predicted locations from SLEAP (**left column**) and observed locations recorded in ZooMonitor (**right column**) for two focal spider monkeys. Observed locations are plotted over a schematic map of the study area, while predicted locations are plotted over video frames from the corresponding date. The blue and red lines split the enclosure into regions with the climbing tree as the center. Yellow and red colors represent increasing location density, with red indicating the highest density. Dates are shown above each pair of panels. Heatmaps of predicted (**left column**) and observed (**right column**) locations for each sampling date, as described in the previous page.

Spatial activity patterns of the two spider monkeys within the indoor enclosure were visualized using heatmaps generated from both manual observations (ZooMonitor) and automated pose estimation (SLEAP) (Figure 2). A total of 12 heatmaps are presented: six pairs corresponding to six observation dates. Each pair displays the manually scored top-down heatmap alongside the automatically generated front-facing heatmap from SLEAP, arranged side by side to facilitate direct comparison.

**Figure A1:**
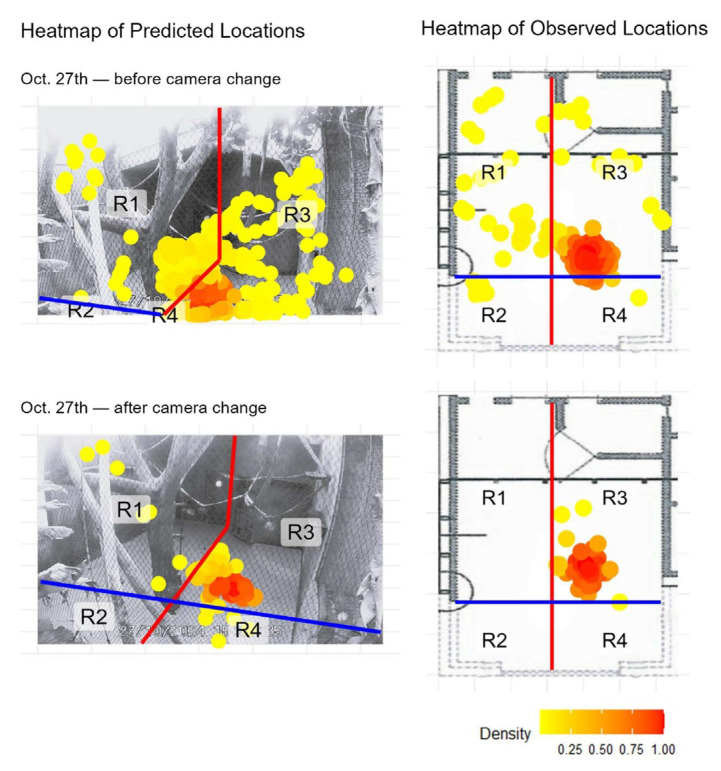
Heatmaps of model-predicted locations from SLEAP (**left column**) and observed locations recorded in ZooMonitor (**right column**) from 27 October, before and after camera angle shift. Observed locations are plotted over a schematic map of the study area, while predicted locations are plotted over video frames from the corresponding date. The blue and red lines split the enclosure into regions with the climbing tree as the center. Yellow and red colors represent increasing location density, with red indicating the highest density.

Overall, the heatmaps from both methods revealed similar spatial use patterns, with high-occupancy zones consistently centered around the enclosure’s central climbing structures and the door with feeding gutters. To facilitate direct comparison, the enclosure was divided into four regions (R1–R4), and the distribution of locations across these regions is summarized in Table A1. Comparison between the methods showed a high degree of agreement for most days, with percentage overlap values ranging from 44.75% to 99.00% and Pearson correlation coefficients: 0.237 ≤ r ≤ 1.000 across observation days (Table 1). Minor discrepancies appeared on some observation dates, most notably on October 27th, where a major discrepancy was found. Further inspection revealed that a major camera shift occurred partway through that day, which affected the alignment of the two methods. To account for this, the data was split into pre- and post-shift segments (Figure A1, Table A2 and Table A3). When analyzed separately, the overlap between methods was 95.02% and 97.51%, with r = 0.99 and r = 1.00, respectively.

**Figure 3:**
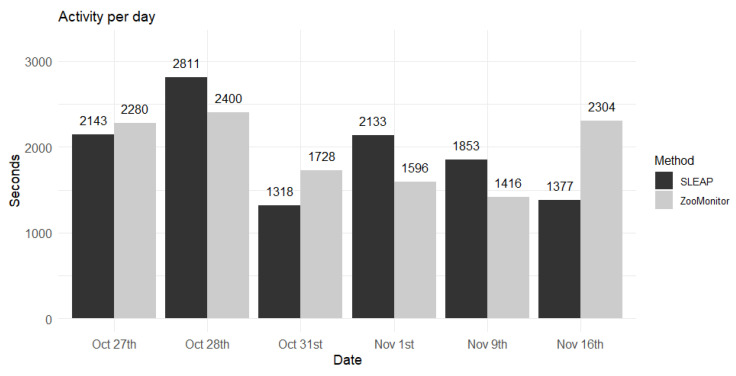
Comparison of activity (seconds in movement) measured with ZooMonitor and SLEAP on 6 non-consecutive days. Bars show the sum of seconds where each spider monkey was in movement according to each method. The numbers on top of the bars indicate the observed value.

The analysis of activity calculated from the tracking results from the two methods, SLEAP and ZooMonitor, generally produced comparable estimates of the individuals’ activity across the observed days (Figure 3). On some days, the ZooMonitor method measured higher activity than SLEAP (27 October, 31 October and 16 November), while the SLEAP method measured higher activity on other days (28 October, 1 November, 9 November). The paired *t*-test resulted in no significant difference between the two methods (t = 0.062, df = 5, *p*-value = 0.952).

## 4. Discussion

This study compared manual enclosure-use tracking with automated pose estimation using SLEAP to evaluate spatial activity in a mother–son pair of spider monkeys. Our results demonstrate a strong overall agreement between methods, supported by high overlap percentages and correlation coefficients, which indicates that computer vision can reliably capture enclosure-use patterns that are traditionally assessed through direct observation. Importantly, both methods identified the same hotspots around the central climbing structures and the door with feeding gutters.

A further dimension of comparison was overall activity levels (Figure 3). Here, both methods produced similar estimates of time spent in movement, with only slight differences between them. Although daily variation occurred, sometimes ZooMonitor estimated higher activity and sometimes SLEAP did, the lack of systematic bias suggests that automated tracking can reliably replicate activity measures. Because both inactivity and abnormal locomotion, such as stereotypic pacing, can signal compromised welfare, these results strengthen the case for computer vision as a valid monitoring tool.

A major advantage of pose estimation was the ability to generate fine-scale, continuous data that minimized observer bias and revealed subtle positional shifts not easily detected with interval sampling. By contrast, manual heatmaps tended to produce more diffuse activity zones, reflecting both the coarser temporal resolution and the subjective estimation of the placement of points on schematic maps. Furthermore, manual scoring remains limited by observer fatigue and reaction time. This highlights the potential for automated methods to provide a more precise and scalable approach to welfare monitoring, especially in settings where large datasets are required.

Despite the overall strong agreement, some discrepancies occurred, highlighting that camera placement and perspective can strongly influence outcomes and must be carefully managed in future applications. Broader coverage, multiple angles, or automated correction methods could help mitigate such issues. Additionally, pose estimation accuracy can be affected by factors such as lighting conditions or temporary occlusions. However, training models on diverse examples and environmental conditions can substantially improve robustness to these challenges.

The findings also raise the question of whether manual tracking remains necessary when reliable automated tools are available. While SLEAP successfully reproduced enclosure-use patterns and overall activity levels, manual observations still hold value for recording nuanced behaviors, social interactions, and context-specific welfare indicators that pose estimation cannot yet capture alone. Future work should therefore integrate pose-based behavior classification and multi-individual tracking, extending beyond space use to assess how animals interact with their environment, such as enrichment. As demonstrated by Hayden et al. (2022) [17], who successfully used pose estimation to quantify specific behaviors in primates. Such extensions would allow welfare monitoring, through computer vision, to move beyond spatial measures toward richer behavioral profiles. While our results demonstrate that automated pose estimation can replicate enclosure-use patterns and activity levels in this case study, further research is needed to assess its generalizability across species, individuals, and settings.

In summary, our results demonstrate that automated pose estimation has the potential to serve as a valid and efficient alternative to manual enclosure-use monitoring. While manual methods may remain important for capturing behavioral nuance, computer vision provides scalable, objective data that can strengthen welfare assessments by enabling continuous, fine-grained monitoring of activity and enclosure use.

## 5. Conclusions

This study demonstrates that computer vision, using pose estimation with SLEAP, can effectively replicate spatial tracking data obtained through manual observations in ZooMonitor. Both enclosure-use patterns and activity levels were captured consistently, highlighting automated tracking as a reliable and scalable method for monitoring welfare-relevant indicators in spider monkeys housed in Aalborg Zoo. Manual observations remain valuable for capturing behavioral nuance, but integrating AI-based methods can enhance the efficiency, consistency, and resolution of welfare assessments. Future work should expand on behavior classification and test generalizability across individuals, environments, and contexts to fully realize the potential of computer vision in zoo-based welfare monitoring.

## Figures and Tables

**Table 1:** Percentage overlap and Pearson correlation (r) between observed locations recorded in ZooMonitor and model-predicted locations from SLEAP across observation dates.

Date	Overlap (%)	Pearson Correlation (r)
27 October	44.75%	0.24
28 October	90.94%	0.99
31 October	82.78%	0.93
1 November	95.37%	1.00
9 November	99.00%	1.00
16 November	83.83%	0.95

## Data Availability

The data presented in this study are available on request from the corresponding author.

## Appendix A

### Regional Distribution Table for SLEAP and ZooMonitor

**Table A1:** Number of observations (n) and corresponding percentages (%) of spider monkey locations in each region (R1–R4) for the SLEAP and ZooMonitor method across observation dates.

Date	Method	R1 (n)	R1 (%)	R2 (n)	R2 (%)	R3 (n)	R3 (%)	R4 (n)	R4 (%)
27 October	SLEAP	131	6.16	6	0.28	780	36.71	1208	56.85
	ZooMonitor	30	12.00	6	2.40	210	84	4	1.60
28 October	SLEAP	314	12.78	0	0.00	2136	86.94	7	0.28
	ZooMonitor	10	3.58	0	0.00	266	95.34	3	1.08
31 October	SLEAP	568	27.32	3	0.29	1492	71.77	16	0.77
	ZooMonitor	82	41.41	5	2.53	108	54.55	3	1.52
1 November	SLEAP	147	7.84	0	0.00	1717	91.53	12	0.64
	ZooMonitor	22	8.24	0	0.00	232	86.89	13	4.87
9 November	SLEAP	937	45.60	6	0.29	1109	53.97	3	0.15
	ZooMonitor	62	44.60	1	0.72	75	53.96	1	0.72
16 November	SLEAP	1225	65.79	6	0.32	623	33.46	8	0.43
	ZooMonitor	170	79.44	6	2.80	37	17.29	1	0.47

## Appendix B

### Appendix B.2. Overlap and Correlation

**Table A2:** Percentage overlap and Pearson correlation (r) between observed locations recorded in ZooMonitor and model-predicted locations from SLEAP from 27 October before and after camera angle shift.

Date	Overlap (%)	Pearson Correlation (r)
27 October before camera change	95.02	0.99
27 October after camera change	97.51	1.00

### Appendix B.3. Regional Distribution Table for SLEAP and ZooMonitor

**Table A3:** Number of observations (n) and corresponding percentages (%) of spider monkey locations in each region (R1–R4) for the SLEAP and ZooMonitor method from 27 October before and after camera angle shift.

Date	Method	R1 (n)	R1 (%)	R2 (n)	R2 (%)	R3 (n)	R3 (%)	R4 (n)	R4 (%)
27 October before camera change	SLEAP	255	16.79	0	0.00	1264	83.21	0	0.00
	ZooMonitor	30	14.93	6	2.99	161	80.10	4	1.99
27 October after camera change	SLEAP	10	1.66	0	0.00	588	97.51	5	0.83
	ZooMonitor	0	0.00	0	0.00	49	100	0	0.00
